# Characterization of Theabrownins Prepared From Tea Polyphenols by Enzymatic and Chemical Oxidation and Their Inhibitory Effect on Colon Cancer Cells

**DOI:** 10.3389/fnut.2022.849728

**Published:** 2022-03-15

**Authors:** Xiaoqiang Chen, Yuxi Hu, Bingjie Wang, Yin Chen, Yao Yuan, Weilong Zhou, Wei Song, Zhengqi Wu, Xiuting Li

**Affiliations:** ^1^National “111” Center for Cellular Regulation and Molecular Pharmaceutics, Key Laboratory of Fermentation Engineering (Ministry of Education), Hubei Key Laboratory of Industrial Microbiology, Hubei University of Technology, Wuhan, China; ^2^Beijing Advanced Innovation Center for Food Nutrition and Human Health, Beijing Technology and Business University (BTBU), Beijing, China; ^3^National Center for Tea Quality Inspection and Testing, Hangzhou Tea Research Institute, All China Federation of Supply and Marketing Cooperatives, Hangzhou, China; ^4^Laboratory of Nutritional and Healthy Food-Individuation Manufacturing Engineering, Research Center of Food Safety Risk Assessment and Control, College of Food Science and Technology, Northwest University, Xi'an, China

**Keywords:** theabrownins, enzyme, characterization, colon cancer cell, inhibition

## Abstract

Theabrownins (TBs) are prepared from dark tea and contain a large number of complex heterogeneous components, such as carbohydrates, proteins, and flavonoids, which are difficult to remove. In addition, some toxic and harmful extraction solvents are used to purify TBs. These obstacles hinder the utilization and industrialization of TBs. In this study, tea polyphenols were used as substrates and polyphenol oxidase and sodium bicarbonate (NaHCO_3_) were used successively to prepare theabrownins (TBs-E). The UV-visible characteristic absorption peaks of the TBs-E were located at 203 and 270 nm and Fourier-transform IR analysis showed that they were polymerized phenolic substances containing the hydroxy and carboxyl groups. The TBs-E aqueous solution was negatively charged and the absolute values of the zeta potential increased with increasing pH. A storage experiment showed that TBs-E were more stable at pH 7.0 and in low-temperature environments around 25°C. HT-29 human colon cancer cells were used to evaluate the biological activity of TBs-E through 3-(4,5)-dimethylthiahiazo (-z-y1)-3,5-di- phenytetrazoliumromide (MTT), H&E staining, propidium iodide immunofluorescent staining, flow cytometry, and real-time PCR assays. The TBs-E significantly inhibited cell growth and caused late apoptosis, particularly at the dose of 500 μg/ml. The TBs-E markedly reduced the expression of antioxidant enzyme genes and increased the generation of reactive oxygen species to break the redox balance, which may have led to cell damage and death. These results will promote research and industrialization of TBs.

## Introduction

Cancer is the first or second leading cause of premature death in nearly 100 countries around the world, while colon cancer is one of the highest rates of cancer morbidity and mortality globally, with an estimated 881,000 deaths in 2018 alone, accounting for one-third of cancer morbidity and mortality globally, along with lung cancer and female breast cancer (1). With advances in medical treatment, new surgical techniques and chemotherapy have improved survival rates for patients with colon cancer (2), while patients suffer great physical and emotional pain. The development of foodborne natural products with anticolon cancer activity is of great value for the prevention and nutritional intervention of patients' prognosis.

Theabrownins (TBs) are major bioactive components in dark teas, such as Pu'er tea, Chin-brick tea, Fu-brick tea, and Liubao tea. TBs are water-soluble polyphenol polymers oxidized from catechins and their gallate derivatives during the dark tea fermentation process. TBs have been extracted from dark tea and their biological activity has been explored. Many studies have reported that TBs contain a large amount of complex heterogeneous components, namely, carbohydrates, proteins, flavonoids, and others (3–7), but some studies did not describe the chemical composition. Moreover, some harmful organic solvents, such as chloroform, ethyl acetate, and n-butanol, are used to separate TBs (8). The preparation process is environmentally unfriendly and harmful to human health. Our unpublished research determined that these heterogeneous components cannot be separated from TBs even after extraction with several organic solvents. These obstacles hinder the utilization and industrialization of TBs. Thus, the development and utilization of TBs are insufficient, even though they have been proven to have good health effects.

In this study, tea polyphenols were used as substrates and polyphenol oxidase (PPO) and sodium bicarbonate (NaHCO_3_) were used to prepare theabrownins (TBs-E). At present, TBs is still in the research stage and it is extracted and separated from dark tea. The preparation of tea polyphenols by the enzymatic or chemical method is rarely reported. Compared with extracting TBs from dark tea as reported previously (8), the enzymatic reaction to prepare TBs is a simple and environmentally friendly process that does not require toxic solvents. In addition, the HT-29 colon cancer cell line was used to evaluate the biological activity of TBs-E. The results of this study will promote research and industrialization of TBs.

## Materials and Methods

### Materials and Reagents

The tea polyphenols (purity 98%) were purchased from Taiyo Green Power Corporation Ltd. (Jiangsu, China). The HT-29 human colon cancer cell line was purchased from the Cancer Institute of the Chinese Academy of Medical Science. RPMI-1640 medium (Thermo Scientific Hyclone, Shanghai, China), supplemented with 10% fetal bovine serum (Thermo Scientific Hyclone), penicillin (100 IU/ml), and streptomycin (100 μg/ml), was used for cell culture at 37°C in a humidified atmosphere with 5% CO_2_. All the other analytical reagents used in the experiment were purchased from Sigma-Aldrich (St Louis, Mosby, USA), and the experimental water was ultrapure (Mill-Q, ≥ 18.2 MΩ cm; Millipore, Milton, Massachusetts, USA).

### Theabrownins Prepared From Tea Polyphenols

A 2.00 mg/ml tea polyphenol (purity 98%) aqueous solution was successively oxidized with PPO (pH 6.0, 35°C) and NaHCO_3_ to obtain brown polyphenol oxidized polymers. After the reaction was completed, the solution was filtered through an ultrafiltration membrane with a molecular weight cutoff of 1,000 Da to remove the phosphates and carbonates and the retentate was filtered with an ultrafiltration membrane with a molecular weight cutoff of 30,000 Da to remove the PPO enzyme, which was freeze-dried to obtain a brown powder. The powder was enzymatically catalyzed TBs-E. Under certain conditions, tea polyphenols may be catalyzed by PPO to form theaflavins (TFs). Since there is no authoritative method for TBs content determination at present, this study indirectly calculated the content of TBs in TBs-E by measuring the contents of residual catechins and caffeine and the content of TFs that may be generated. The contents of residual catechins and caffeine in the TBs-E were determined by high-performance liquid chromatography (HPLC) according to the national standards GB/T8313-2018 as well as TFs determined using the HPLC method GB/T 30483-2013. The total content of residual catechins in the TBs-E was 2.11%, and caffeine content was 0.10%. There were no TFs detected in TBs-E. The calculation formula for the content of TBs in TBs-E was calculated as 1 – (*C*_remainingcatechins_ + *C*_caffeine_ + *C*_TFs_)/(2.00 × 98%). The content of TBs in TBs-E was 95.89%.

### Sodium Dodecyl Sulfate-Polyacrylamide Gel Electrophoresis (SDS-PAGE) to Determine Whether PPO Was Removed From the TBs-E

A multistage ultrafiltration membrane was adopted to separate and remove the remaining PPO and salt during the enzymatic oxidation of tea polyphenols to prepare the TBs-E. Therefore, it was necessary to test whether the enzyme protein was in the TBs-E. SDS-PAGE was used to detect the residual amount of enzyme in TBs-E. A 5.00% concentrated gel and 12.00% separation gel were prepared for 70 V constant-voltage electrophoresis. When the sample reached the boundary between the separation gel and the concentrated gel, the current was adjusted to 110 V until the end of electrophoresis (9).

### Chrominance of the TBs-E Aqueous Solution and Solid Powder

The chrominance analysis of the TBs-E dissolved in deionized water (2.00 mg/ml) was performed using a Minolta Colorimeter (CM-3500d) and the L^*^, a^*^, and b^*^ values were determined (10). The chroma analysis of the TBs-E solid powder was performed using a colorimeter (ColorMeter MAX), and the L^*^, a^*^, and b^*^ values were determined (10).

### Ultraviolet-Visible (UV-Vis) Spectroscopy and Fourier-Transform IR (FTIR) Spectroscopy

The UV-visible (UV-vis) scanning spectra of 10.00 μg/ml TBs-E and tea polyphenol aqueous solutions were recorded using a TU-1900 spectrophotometer (Persee, Beijing, China) in the 200–700 nm region (11).

The FTIR spectra of the TBs-E and tea polyphenols were recorded on a Nicolet Nexus 670 FT-IR spectrometer (Brook Instruments Incorporation, Dresden, Germany), with an average of 32 scans at 4 cm^−1^ resolution from 4,000 to 400 cm^−1^ (12).

### Zeta Potential of the TBs-E Aqueous Solution as a Function of pH

The TBs-E were dissolved in ultrapure water (0.5 mg/ml). The zeta potential of TBs-E was determined with a Zetasizer Nano-ZS particle diameter and potentiometric analyzer. The pH of the TBs-E aqueous solution was adjusted from 3.0 to 9.0 with solutions of 0.1 M hydrochloric acid and 0.1 M sodium hydroxide solution at an interval of 1.0 and a temperature of 25°C (13, 14).

### Scanning Electron Microscopy (SEM)

Scanning electron microscopy (Japan NTC JSM-6390LV) was used to observe the microscopic morphology of the TBs-E. The TBs-E solid powder was glued on a sample stage with conductive carbon, sprayed with gold (50 nm) and detected at an accelerating voltage of 4–5 kV (15).

### Stability of the TBs-E Aqueous Solution Under Different pH and Temperature Conditions

The TBs-E were dissolved in ultrapure water (1.00 mg/ml). The pH of the suspension was adjusted from 3.0 to 9.0 with solutions of 0.1 M hydrochloric acid and sodium hydroxide at an interval of 1.0 and a temperature of 25°C. A UV-2550 spectrophotometer (Shimadzu) was used to record the absorbance of the TBs-E aqueous solutions at 600 nm every 24 h for a total of 5 days.

The TBs-E was dissolved in ultrapure water to prepare samples of the same concentration (1.00 mg/ml). The TBs-E aqueous solution was heated in constant temperature water baths at 25, 50, and 90°C, respectively. A UV-2550 spectrophotometer was used to record the absorbance of the TBs-E aqueous solution at 600 nm every 2 h for 8 h.

### Effect of the TBs-E on Colon Cancer Cells

#### Cell Inhibition Assay

The inhibitory effect of the TBs-E was investigated using the 3-(4,5)-dimethylthiahiazo (-z-y1)-3,5-di- phenytetrazoliumromide (MTT) assay. The HT-29 (1.00 × 10^5^ cells/ml) was seeded in 96-well culture plates for a 24-h incubation. The TBs-E fine powder was dissolved in a cell culture medium and filtered through a 0.22-μm membrane. A series of solutions containing TBs-E (100, 250, 500, 800, and 1,000 μg/ml) were incubated for 24, 48, or 72 h, respectively. The cells were washed with phosphate buffer saline (PBS) and the MTT solution (0.5 mg/ml) was added to the wells for a 4-h incubation. The MTT was removed and dimethyl sulfoxide (DMSO) was added to dissolve the formazan dye precipitate. The plates were shaken for 10 min. The absorbance values were measured with a plate reader (Bio-Rad-500, Bio-Rad Laboratories Incorporation, Hercules, California, USA) at 490 nm. The inhibitory rate was calculated as $\left(1-\frac{A_{Sample}}{A_{Control}}\right)\times \;100\%$.

#### Hematoxylin and Eosin Staining

Hematoxylin and eosin staining were used to observe the morphology of the HT-29 cells after the 500 μg/ml TBs-E treatment. The cells were seeded in 12-well culture plates with carry sheet glass at an initial density of 1 × 10^5^ cells/ml for 24, 48, and 72 h incubations. H&E staining was used to detect the morphological changes by conventional methods and the sections were dehydrated through a graded ethanol series, cleared in xylene, observed with a microscope (EVOS XL Core, Thermo Fisher Scientific, Waltham, Massachusetts, USA), and documented by photography.

#### Propidium Iodide (PI) Immunofluorescent Staining

After treatment with or without 500 μg/ml TBs-E for different time intervals, cells were seeded in 12-well plates with carry sheet glass and washed with PBS. The 50 μg/ml PI solution was mixed with the cells at room temperature in the dark. After washing with PBS, a fluorescence microscope (ZEISS, Axio vert. A1, Jena, Germany) and a digital charge-coupled device were applied to collect fluorescence images.

#### Apoptosis Detection by Flow Cytometry

HT-29 cells were co-cultured with 500 μg/ml TBs-E in 6-well plates for 48 h. The cells were harvested and washed in precooled PBS, then stained with a solution of Annexin V and PI for 15 min at room temperature according to the kit instructions. The percentage of apoptotic cells was determined by flow cytometry (Beckman Coulter FC500, Beckman, Brea, California, USA) and analyzed with CXP software.

#### Real-Time PCR Analysis

Gene expression of glutathione peroxidase (GSH-PX), catalase (CAT), and superoxide dismutase (SOD) was detected in HT-29 cells treated with TBs-E by real-time PCR. Total RNA was isolated using the EasyPure^TM^ RNA Kit (Sigma-Aldrich). The TransScript® One-Step gDNA Removal and cDNA Synthesis SuperMix Complimentary Kit (Applied Biosystems, Foster City, California, USA) was used to synthesize the cDNA. The primers were designed by Sangong Bioengineering (Shanghai) Corporation Ltd. PCR amplification was performed on the ABI Prism 7300 Sequence Detection System (Applied Biosystems) and fluorescence was monitored in real-time. All the data were normalized to the β-actin internal housekeeping gene. Data were analyzed using the delta-Ct method and represented as mean ± SEM. A *p*-value <0.05 was considered as statistically significant.

#### Intracellular Reactive Oxygen Species (ROS) in HT-29 Cells

Intracellular levels of ROS were detected using the 2',7'-dichloro fluorescein diacetate (DCFH-DA) to fluorescent 2',7'-dichlorofluorescin molecular probe according to the Reactive Oxygen Species Assay Kit Protocol (Beyotime Biotechnology, Shanghai, China). HT-29 cells with an initial density of 5 × 10^5^ cells/ml were treated with 500 μg/ml TBs-E for 48 h in a 6-well plate. The cells were incubated with 10 μM DCFH-DA for 20 min at 37°C in the dark. After being washed two times in cold PBS, the cells were analyzed with a fluorescence microscope (ZEISS, Axio vert. A1) to determine the ROS fluorescence intensity.

#### Statistical Analysis

The statistical analysis was performed with SPSS version 13.0 software (SPSS Incorporation, Chicago, Illinois, USA) and Origin 9.6 software (Origin Laboratories, Northampton, Massachusetts, USA).

## Results

### Removal Effect of PPO in TBs-E

**Figure 1:**
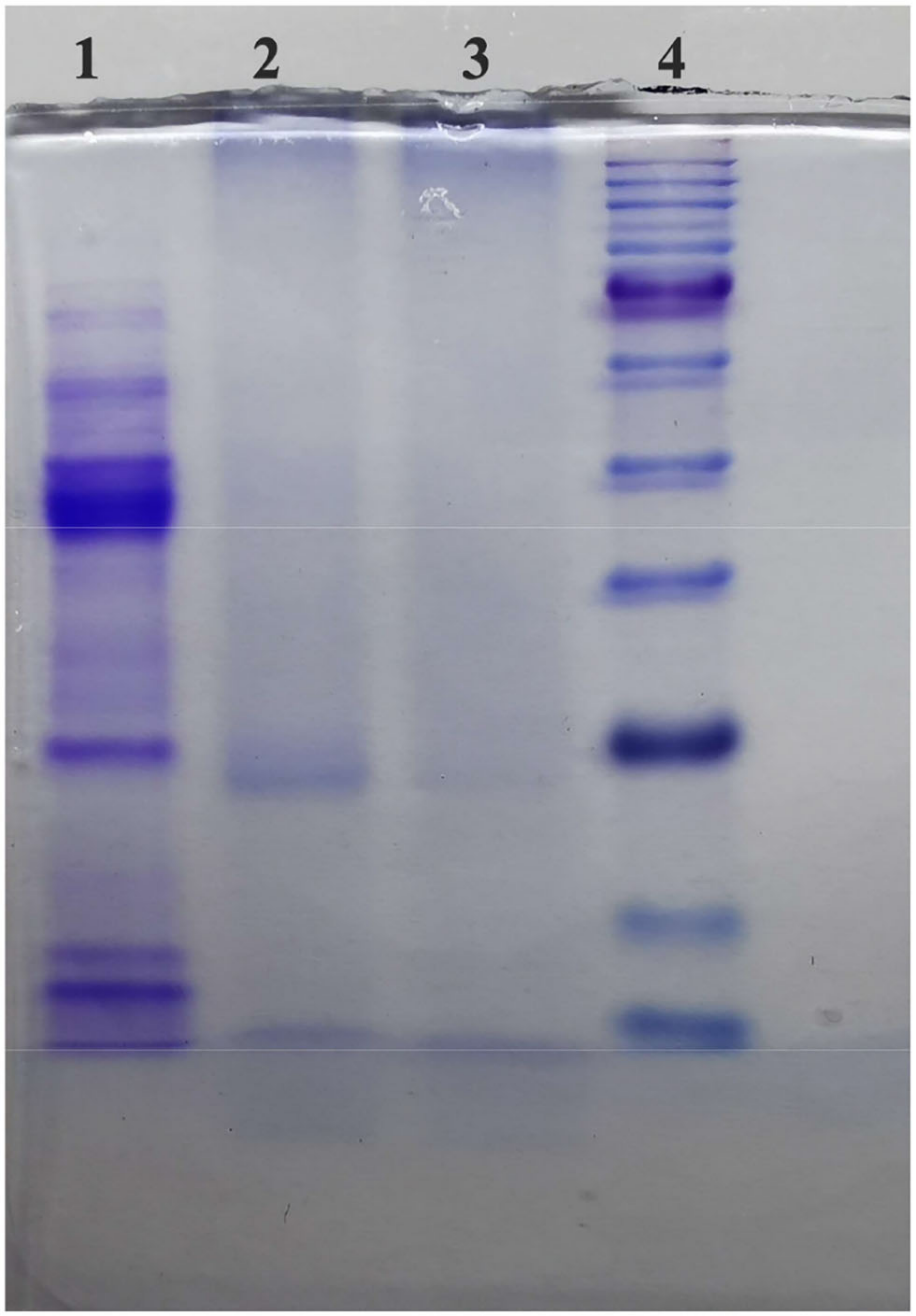
Sodium dodecyl sulfate-polyacrylamide gel electrophoresis (SDS-PAGE) to detect PPO in 2.00 mg/ml theabrownins (TBs-E) (5.00% concentrated gel, 12.00% separation gel, and 70 V constant-voltage electrophoresis). Among the lanes, lane 1 refers to polyphenol oxidase (PPO), lanes 2 and 3 refer to the TBs-E, and lane 4 refers to the marker proteins.

The remaining PPO enzyme in the TBs-E and salt were separated and removed with a multistage ultrafiltration membrane. SDS-PAGE was used to detect whether the enzyme was completely removed from the TBs-E. The results of SDS-PAGE are shown in Figure 1. The marker proteins were a positive control, as shown in bands 1 and 5. The second and fourth bands were the TBs-E electropherograms. The second and fourth bands showed no protein, indicating that there was no residual PPO in the TBs-E sample.

### Chrominance Analysis

The L^*^, a^*^, and b^*^ table color system was used to analyze the TBs-E chroma values. The L^*^, a^*^, and b^*^ values of a 2.1 mg/ml TBs-E aqueous solution were 50.72, 23.35, and 71.86, and those of the TBs-E solid powder were 41.10, 3.75, and 1.78. The L^*^, a^*^, and b^*^ indices indicate the lightness, red-green chroma, and yellow-blue chroma index, respectively. The depth of the color was proportional to the absolute value of a^*^ and b^*^ (11). This result indicates that the 2.0 mg/ml TBs-E aqueous solution and its solid powder had deep yellow-red chroma (also called brownness).

### UV-Vis and FTIR Spectra

**Figure 2:**
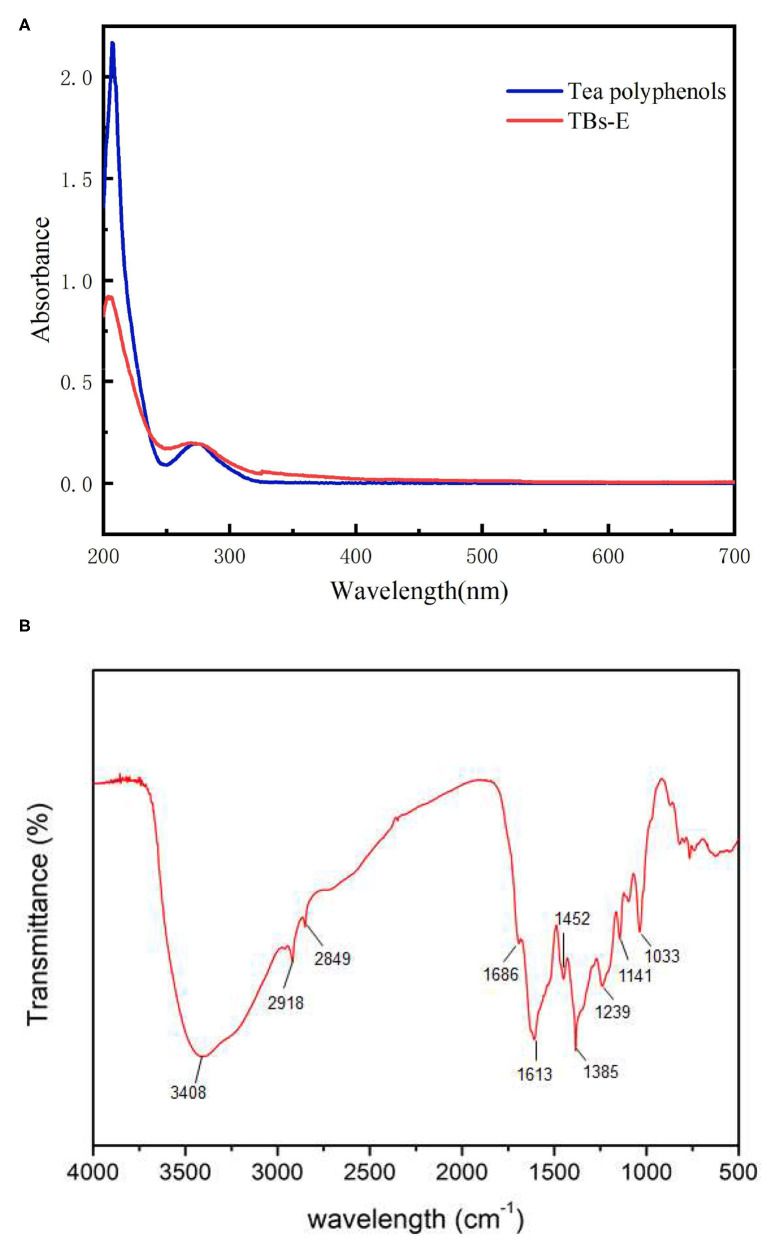
UV-vis spectra of 10.00 μg/ml TBS-E and 10.00 μg/ml tea polyphenols in the 200–700 nm scanning region **(A)**, and the Fourier-transform infrared spectrum of TBs-E in the 500–4,000 cm^−1^ scanning region **(B)**.

UV-vis spectroscopy is widely used for the qualitative and quantitative measurements of inorganic and organic substances (4). As shown in Figure 2A, the UV-vis scan spectra of the TBs-E and tea polyphenols revealed two characteristic absorption peaks at wavelengths of 204 and 270 nm and 207 and 273 nm, respectively. The UV-vis scan spectrum of the TBs-E is largely consistent with the results on the tea polyphenols as well as some previous studies (3–7), although the TBs in those studies contain a mixture of polysaccharides and proteins. Our previous studies have demonstrated that tea polysaccharides containing covalently bound proteins do not have characteristic absorption peaks (10, 11). Therefore, the characteristic absorption peak of TBs in the UV scanning spectrum is not affected by tea polysaccharide conjugates.

The FTIR spectra showed that the TBs-E samples exhibited absorption peaks at about 3,408, 2,918, 2,849, 1,686, 1,613, 1,452, 1,385, and 1,239–1,000 cm^−1^ (Figure 2B). According to FT-IR spectroscopic analysis, the TBs-E peak at 3,408 cm^−1^ is the characteristic absorption peak of O-H tensile vibrations of the polymer (4, 7). The absorption peaks at about 2,918 and 2,849 cm^−1^ are due to C-H stretching vibrations of the CH, CH_2_, and CH_3_ groups (16). The absorption peaks at about 1,686 and 1,611 cm^−1^ are due to C=O stretching vibrations. The absorption peaks at 1,452 and 1,385 cm^−1^ are due to vibrations in the tensile aromatic hydrocarbon C=C, and the absorption peaks in the range of 1,239–1,000 cm^−1^ are due to C-O tensile vibrations (4).

### Zeta Potential as a Function of pH

**Figure 3:**
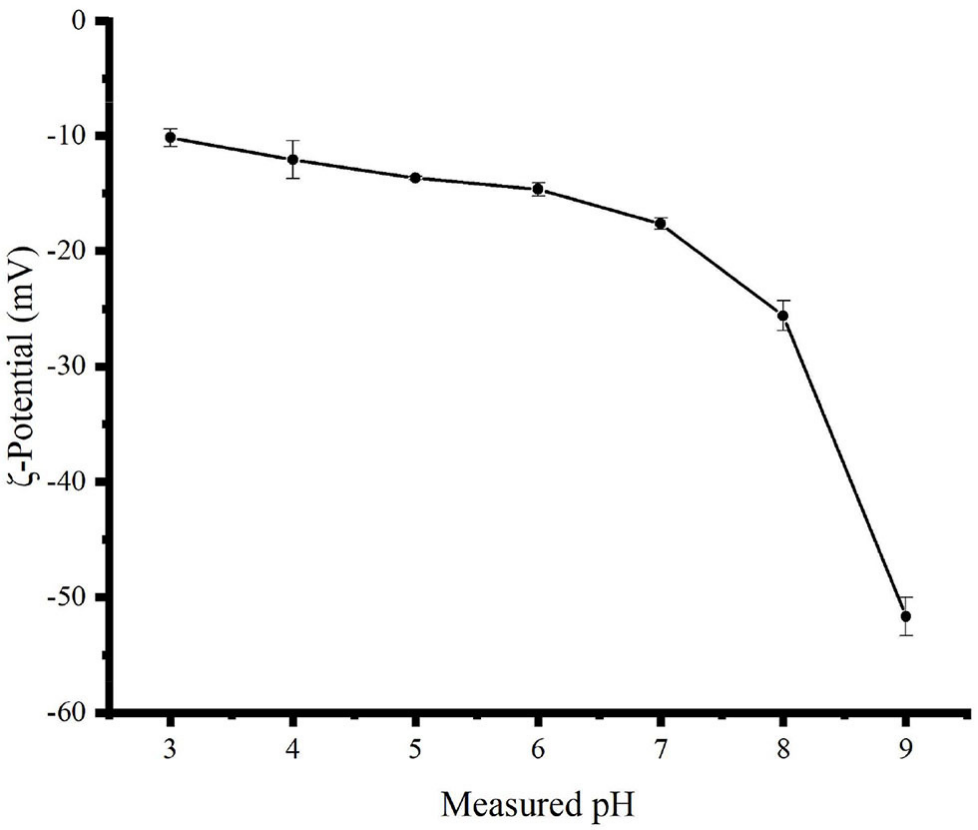
Zeta potential curves of a 0.5 mg/ml TBs-E aqueous solution as a function of pH 3.0–9.0 adjusted using HCl and NaOH at an interval of 1.0 and a temperature of 25°C.

As shown in Figure 3, the zeta potential values of the TBs-E in acidic and alkaline aqueous solutions were negative, indicating an acidic oxidation product (11). When the pH of the solution was changed from 3 to 9, the zeta potential absolute values of TBs-E increased from 10.15 to 51.65 mV. Furthermore, as the pH was increased from 7 to 9, the zeta potential absolute values of the TBs-E varied between 17.60 and 51.65 mV. The greater the absolute value of the zeta potential, the more stable the solution system (11, 17, 18). The TBs-E was less prone to precipitation in aqueous solutions at pH 8 or 9. Alkaline conditions further oxidized TBs-E, resulting in a significant increase in the absolute value of its zeta potential.

### Morphological Characteristics of the TBs-E Powder

**Figure 4:**
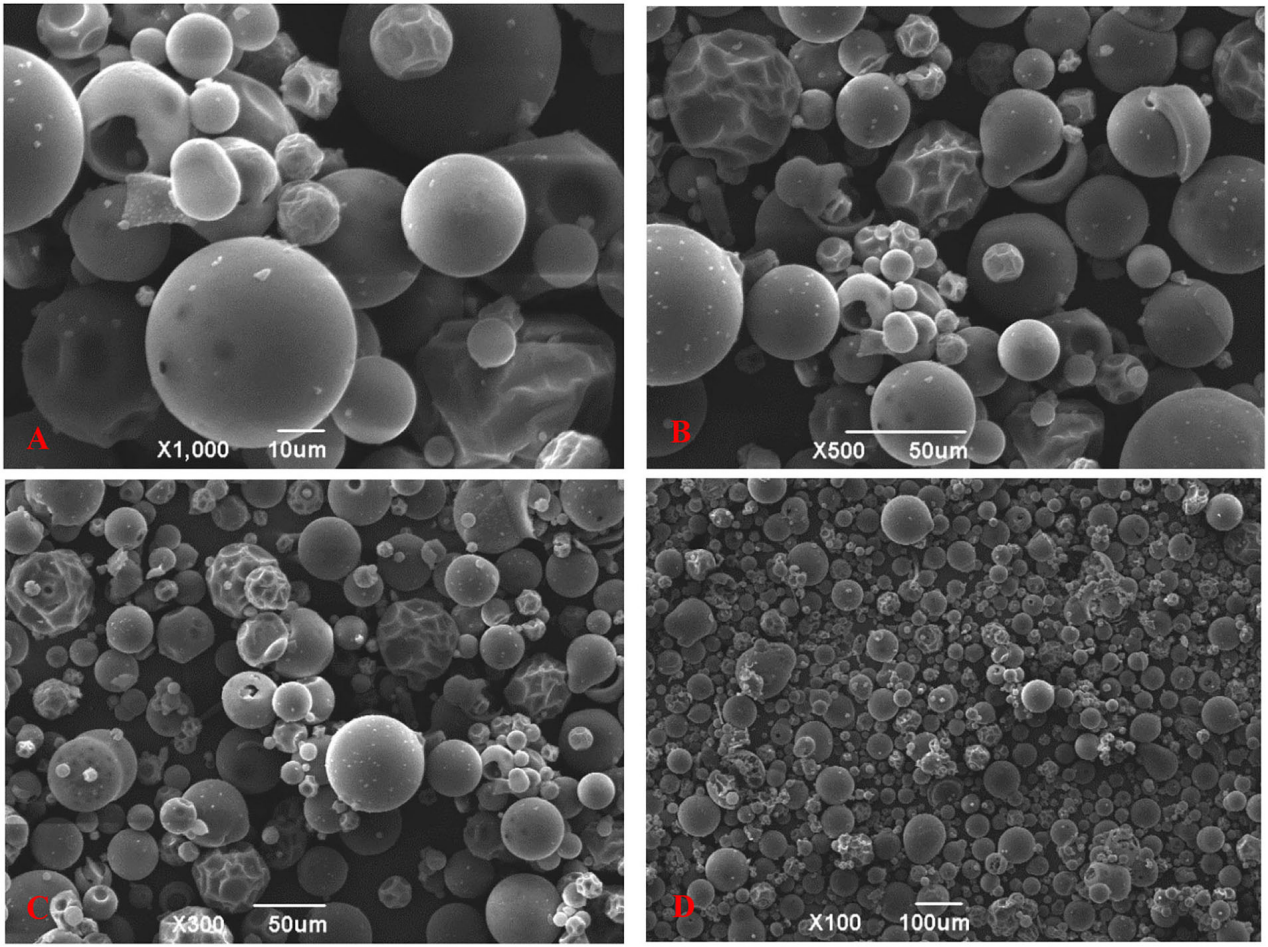
Scanning electron microscopy (SEM) micrographs of TBs-E; **(A)** zoom in 1,000X (10 μm), **(B)** zoom in 500X (50 μm), **(C)** zoom in 300X (50 μm), and **(D)** zoom in 100X (100 μm).

The microscopic surface morphology of the TBs-E is shown in Figure 4. At 100, 300, 500, and 1,000X magnification, most of the TBs-E powder particles were spherical and their surfaces were relatively smooth. A small portion of the TBs-E was wrinkled with depressions on the surface, which was related to the high evaporation of water during the freeze-drying process (19).

### Stability of the TBs-E Aqueous Solution Under Different pH and Temperature Conditions

**Figure 5:**
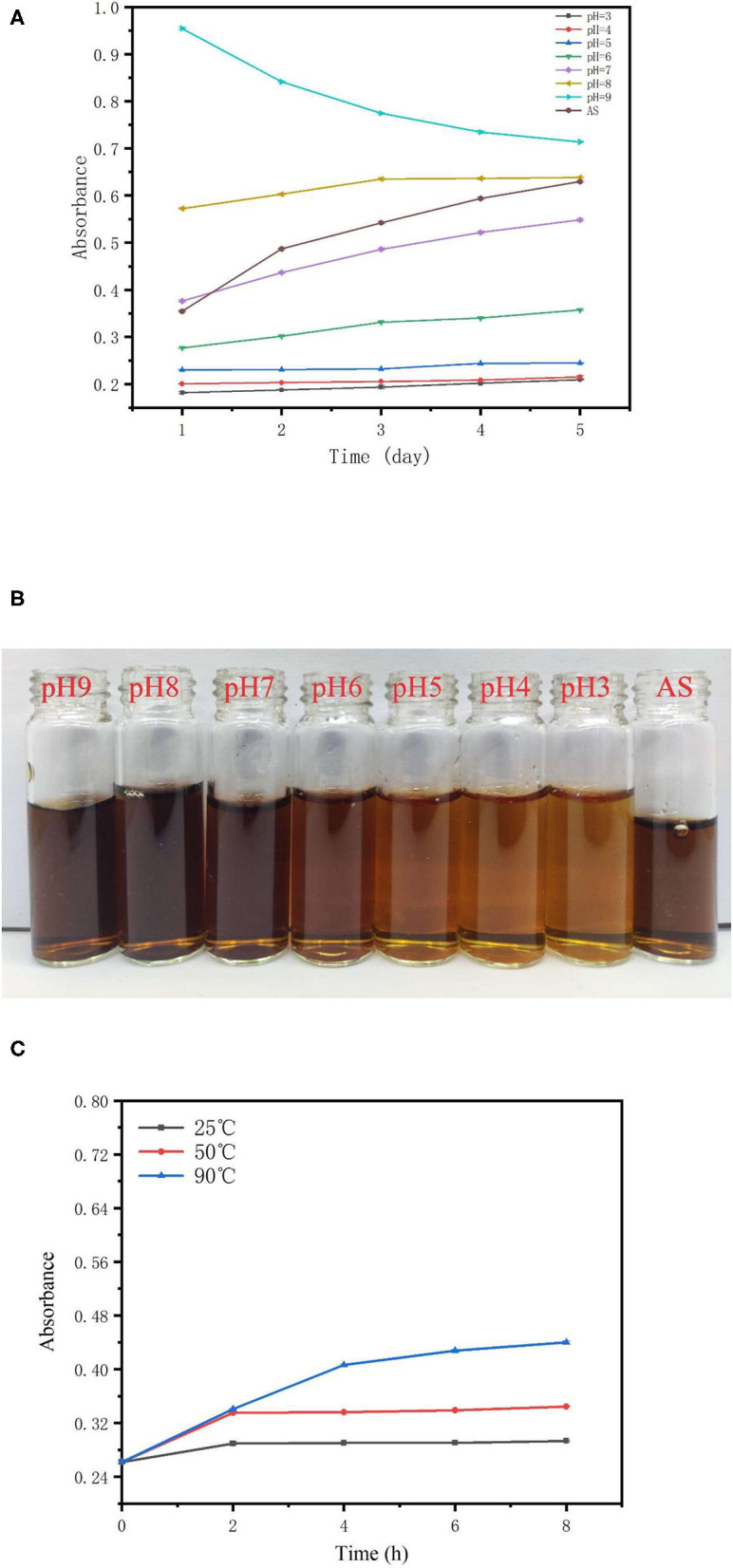
Effects of different pHs and temperatures on the stability of TBs-E. **(A)** Change in the TBs-E absorbance curves in pH 3.0–9.0 solutions and a purified aqueous solution (AS) after 5 days of storage, **(B)** the color of newly prepared TBs-E in different pH and ultrapure aqueous solutions, and **(C)** change in the absorbance curves of TBs-E aqueous solutions stored at 25, 50, and 90°C for 8 h.

In this study, pH 3.0–9.0 was the pH after the TBs-E were dissolved in water and adjusted with acid or alkali. The stability of the TBs-E stored in solutions of different pHs for 5 days is shown in Figure 5A. Among them, AS stands for TBs-E aqueous solution and the AS curve describes the change in absorbance of TBs-E dissolved in ultrapure water for 5 days. The initial absorbance values of the TBs-E in a pH 3.0–9.0 solution environment were 0.182, 0.201, 0.230, 0.277, 0.376, 0.572, and 0.954, respectively. The initial absorbance value of the TBs-E (AS) was 0.355. The higher the pH, the darker the brown color of the TBs-E solution (as shown in Figure 5B), and the higher its absorbance. This observation indicates that TBs-E may undergo various degrees of oxidative polymerization in an alkaline environment.

The absorbance of the TBs-E solution at pH 3.0–5.0 varied very little, indicating that it was stable in a solution of this pH range. The initial absorbance of TBs-E in a pH 3.0–5.0 solution was 0.13–0.17 lower than that of TBs-E (AS) and its color was significantly lighter than that of TBs-E (AS). This finding indicates that the strong acidity of the solution may lead to different degrees of TBs-E degradation. The pH of the TBs-E (AS) was 7.30, and its initial absorbance value was close to that of a pH 7.0 TBs-E solution. After TBs-E (AS) was stored for 5 days, absorbance reached 0.630, which was a 77% increase, and its color deepened, indicating that the TBs-E had undergone further oxidative polymerization during storage.

The TBs-E had the smallest change in absorbance in the pH 8.0 solution during storage, but its initial absorbance was 61% higher than that of TBs-E (AS). The initial absorbance of the pH 9.0 TBs-E solution was 169% higher than that of TBs-E (AS). Absorbance decreased by 25% after 5 days of storage. Thus, the pH 7.0 TBs-E solution was more conducive to long-term storage and stability.

The absorbance value was measured every 2 h to characterize the stability of TBs-E under different temperatures. The change in the absorbance curve of TBs-E stored at 25, 50, and 90°C for 8 h is shown in Figure 5C. The absorbance value of TBs-E increased as the storage temperature was increased. In the first 2 h of storage, the absorbance of the TBs-E aqueous solution at 25 and 50°C increased by 10.67 and 27.86%, respectively, and remained stable for the next 6 h. At 90°C, the absorbance value continued to rise for 8 h, with a linear increase of 54.96% in the first 4 h and then an upward trend that tended to flatten out. In the long-term storage period, the TBS-E is more stable and less prone to oxidative polymerization or decomposition at pH 7.0 and a low temperature around 25°C.

### Inhibitory Effect of TBs-E on Colon Cancer Cells

#### TBs-E Inhibits HT-29 Cell Proliferation

**Figure 6:**
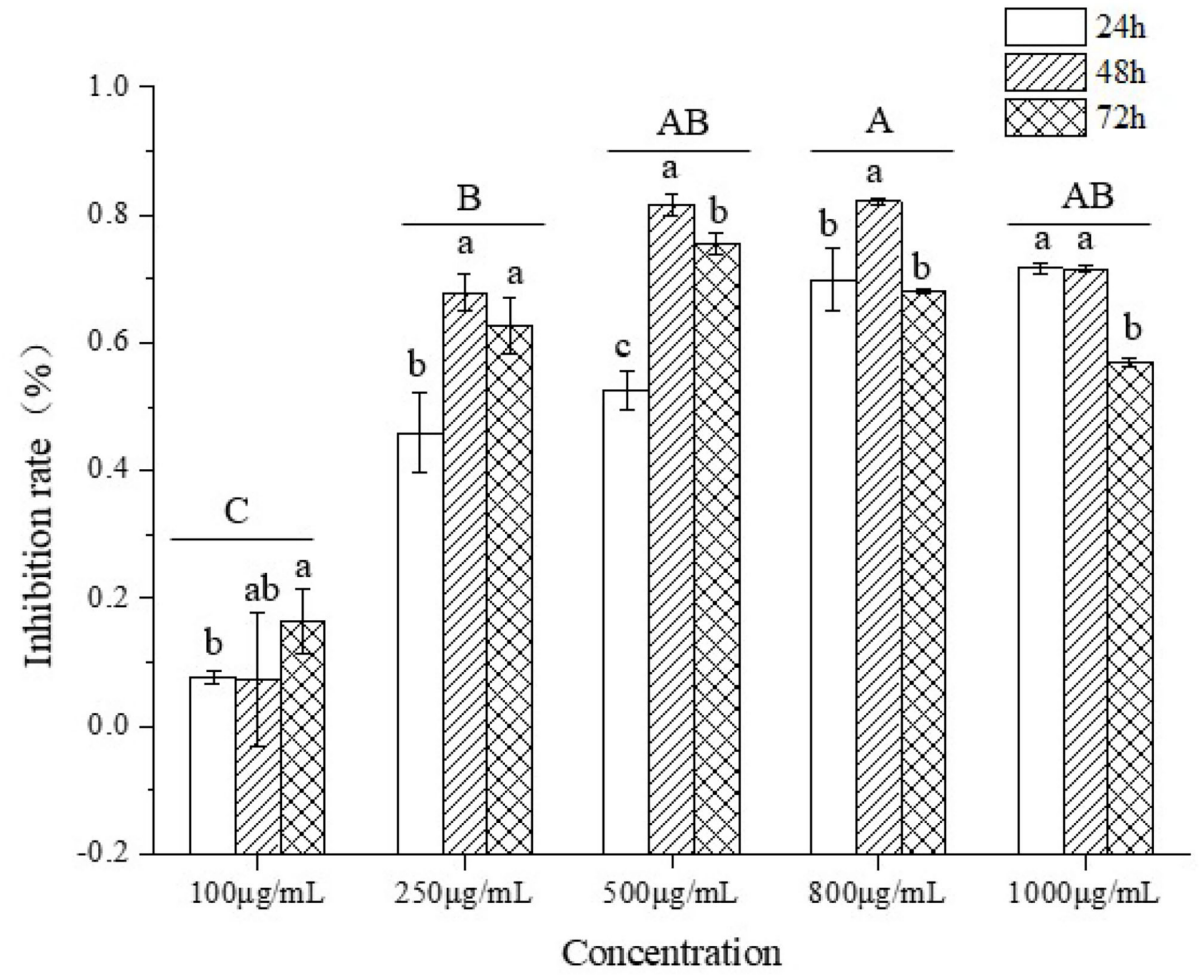
The inhibitory effect on HT-29 cell proliferation.

To test if TBs-E could suppress the proliferation of HT-29 cells, the cells were treated with a series of TBs-E concentrations for 24–72 h. The HT-29 cell proliferation was significantly inhibited, particularly at a dose of 500 μg/ml after 48 h of treatment. No significant increase in the inhibition rate was detected after increasing the concentration to 800 and 1,000 μg/ml. Therefore, 500 μg/ml was used as the dose in subsequent experiments (Figure 6).

#### Cell Morphological Changes After TBs-E Treatment

**Figure 7:**
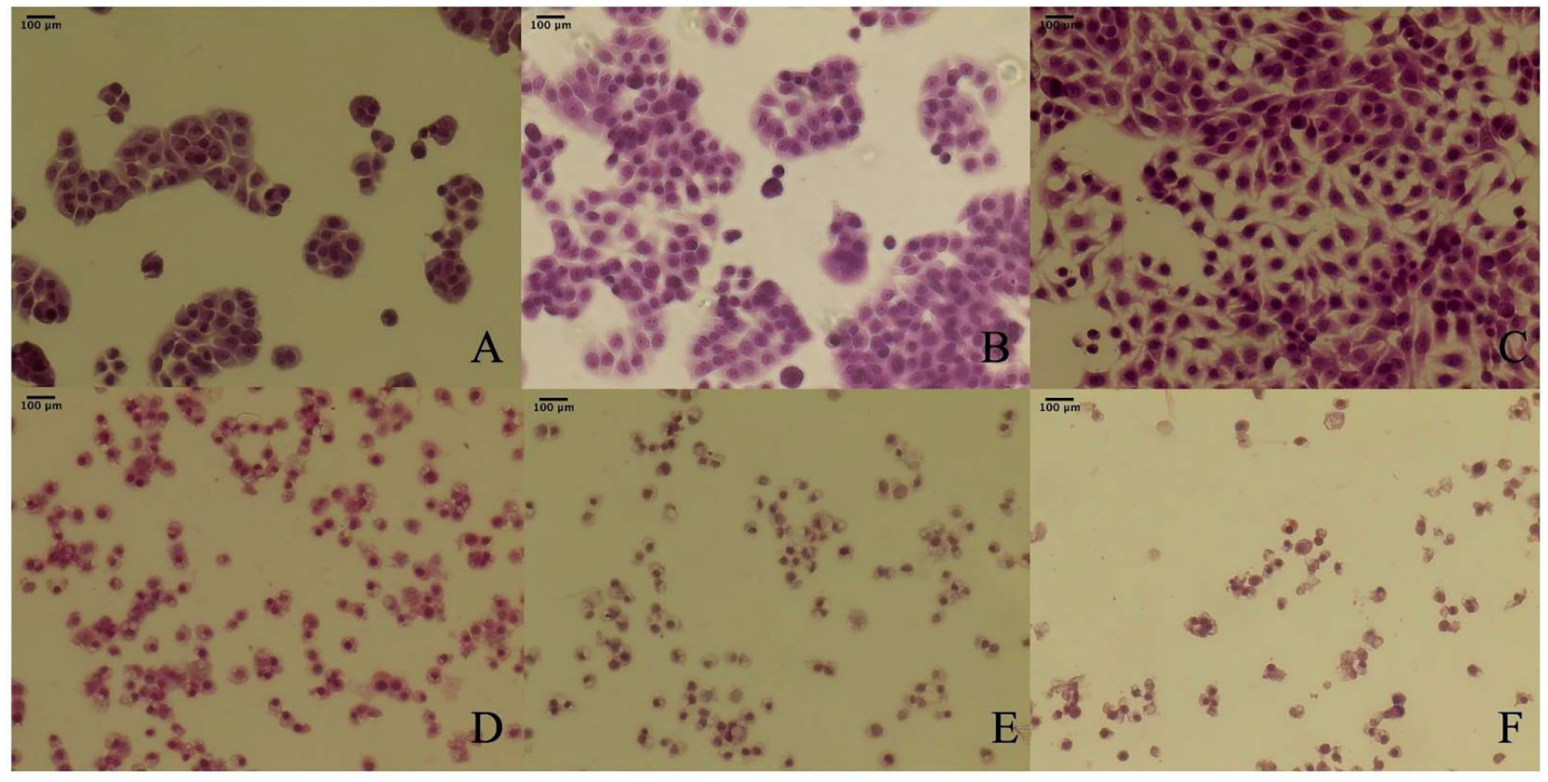
Effect of TBs-E on HT-29 cell morphology (200X); **(A)** control (24 h), **(B)** control (48 h), **(C)** control (72 h), **(D)** TBs-E treatment (24 h), **(E)** TBs-E treatment (48 h), and **(F)** TBs-E treatment (72 h).

The cell morphological changes after the TBs-E treatment were detected by HE staining. The cells were treated with 500 μg/ml TBs-E for 24, 48, and 72 h. Irregular morphology, such as shrinkage of the nucleus, which is typical of apoptosis, was detected after 24 h of treatment. Cellular contents were diminished in most of the cells after 48 h; however, the nucleus was rarely seen in 72 h TBs-E treated cells (Figure 7).

#### Induction of Apoptosis by TBs-E

**Figure 8:**
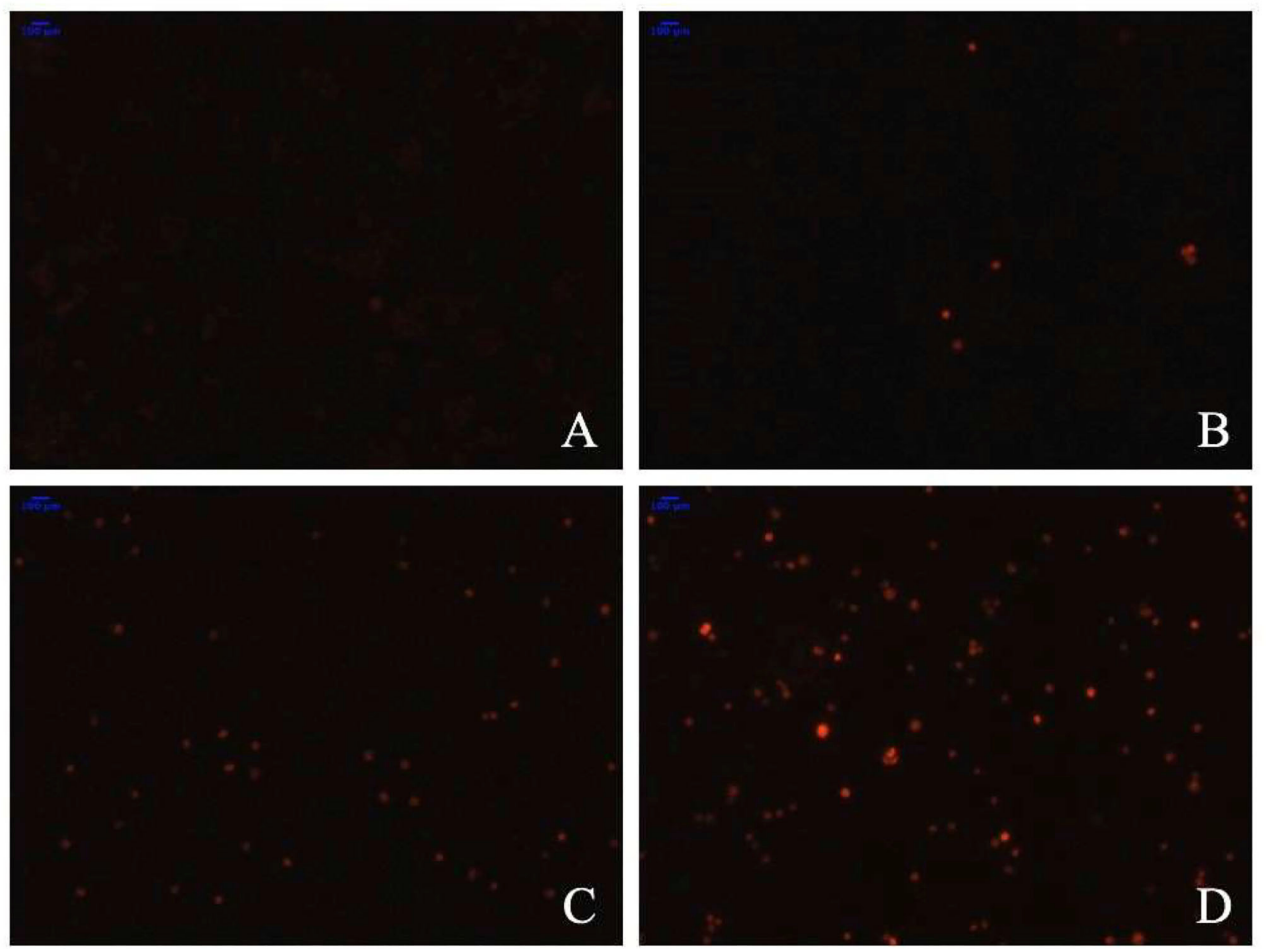
Effect of TBs-E on cell apoptosis (200X); **(A)** control, **(B)** TBs-E treatment (24 h), **(C)** TBs-E treatment (48 h), and **(D)** TBs-E treatment (72 h).

**Figure 9:**
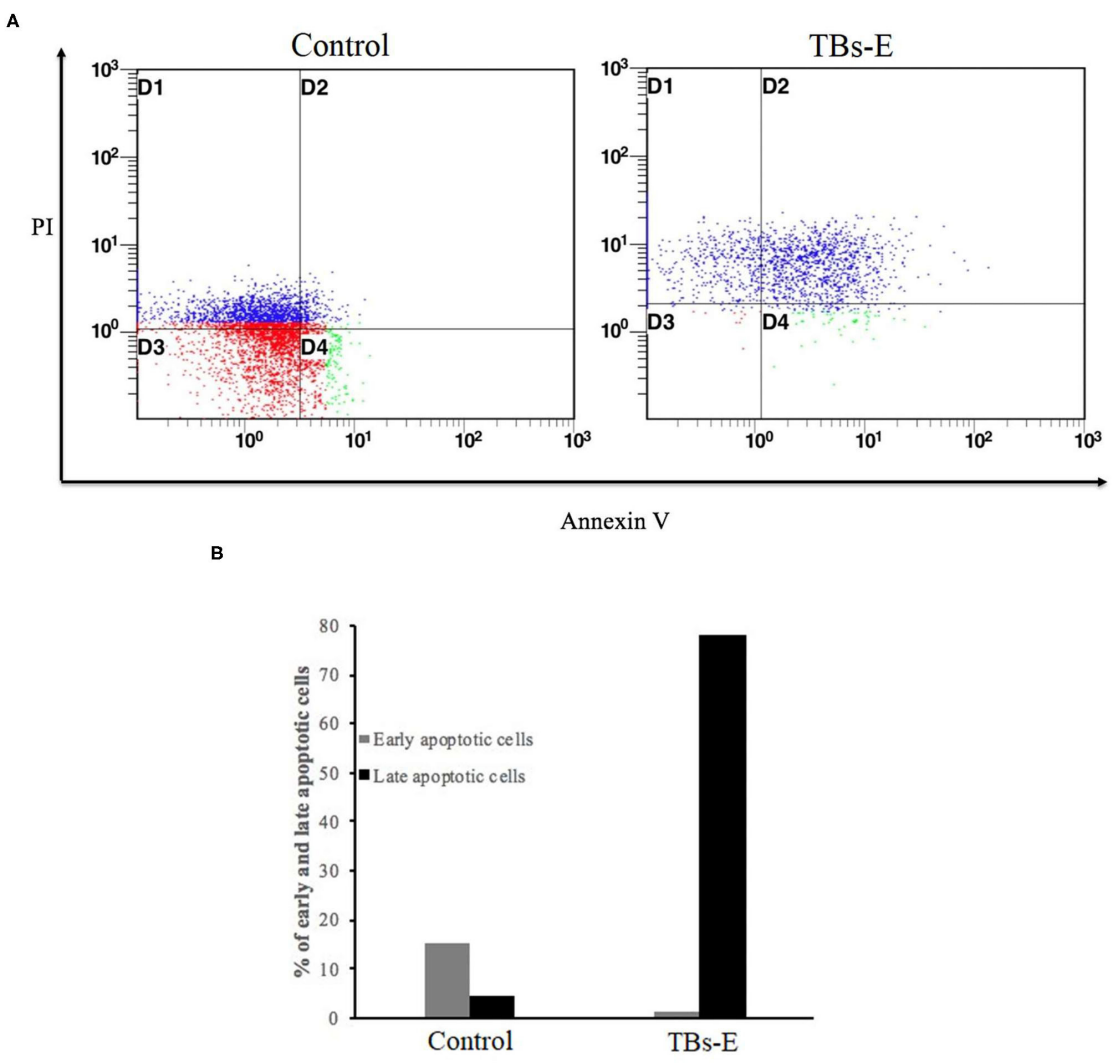
Determination of the apoptotic rate by flow cytometry; **(A)** PI-annexin V flow cytometry to detect cell apoptosis and **(B)** statistical analysis of cell apoptosis.

To determine whether the TBs-E induced HT-29 cell apoptosis, we examined the changes in HT-29 cells after the TBs-E treatment by PI staining. The fluorescence intensity and the number of apoptotic cells increased gradually at different time intervals after the TBs-E treatment compared to the control (Figure 8). The PI results (Figure 8) were consistent with the HE staining results (Figure 7). Flow cytometry was conducted with double-labeled PI and annexin V after a 48 h incubation with TBs-E to determine the rate of cell apoptosis. A total of 78.23% of the cells were in late apoptosis compared to the control (Figure 9), demonstrating that apoptosis or necrosis occurred after the TBs-E treatment.

#### TBs-E Regulates the Antioxidant Enzyme Genes

**Figure 10:**
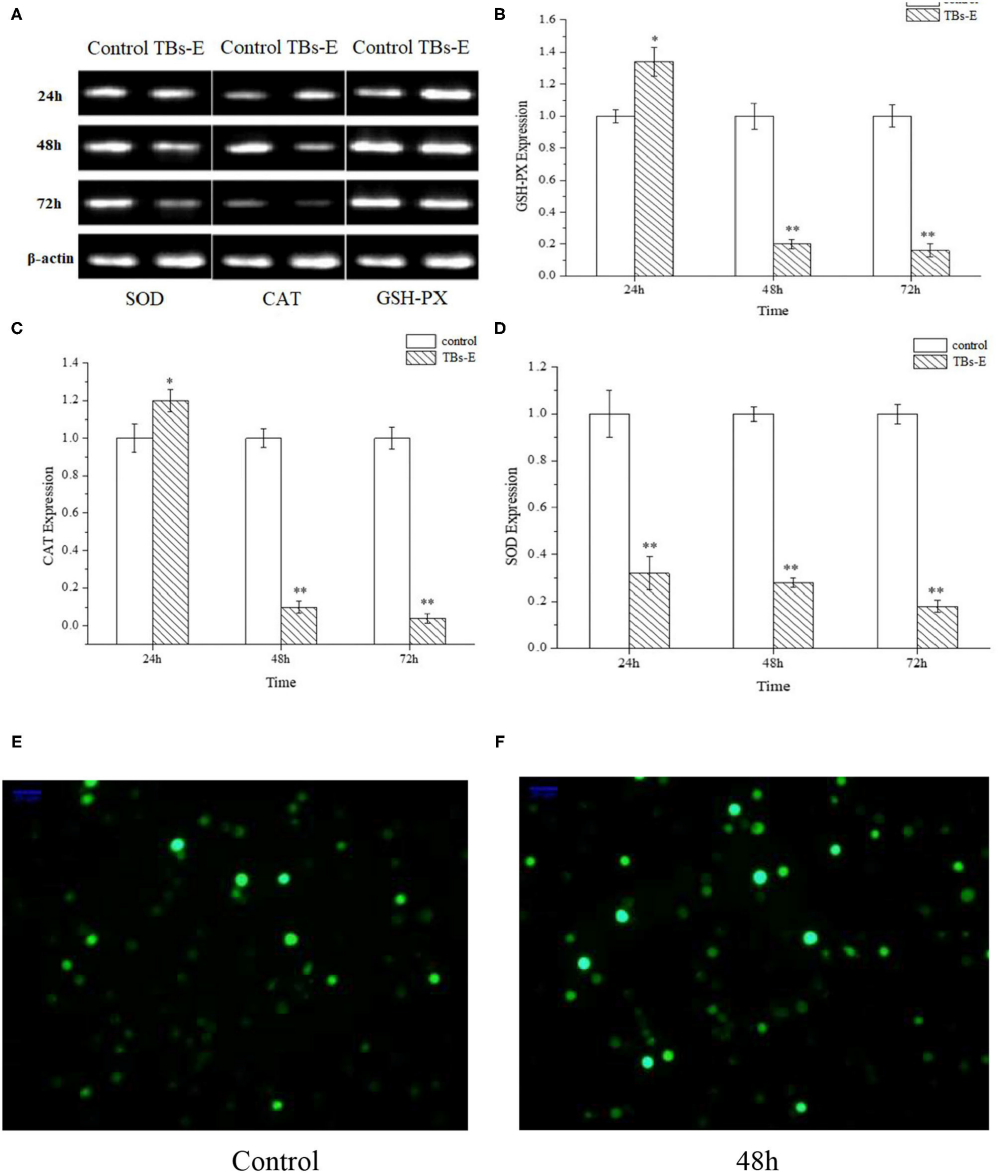
Effects of TBs-E on the expression of the antioxidant enzyme genes in HT-29 cells and immunofluorescence assay to determine ROS in HT-29 cells after the TBs-E treatment (200X). HT-29 cells were treated with 500 μg/ml of TBs-E for 24, 48, or 72 h. After extracting the DNA and synthesizing the cDNA, the final PCR products were subjected to agarose gel electrophoresis **(A)**. The density of the bands was scanned and analyzed by Quantity One software **(B–D)**. *P < 0.05, **P < 0.01, compared to the control group. **(E)** Control, **(F)** TBs-E treatment (48 h).

To further illustrate the underlying mechanism by which TBs-E exerts its inhibitory effects on the proliferation of HT-29 cells, we examined the expression of genes encoding the antioxidant enzymes GSH-PX, CAT, and SOD after treatment with 500 μg/ml TBs-E for 24 h. As result, GSH-PX and CAT gene expression increased significantly (*P* < 0.05). However, the expression levels of both genes decreased significantly (*P* < 0.01) as the treatment time was extended to 48 and 72 h. SOD gene expression was effectively suppressed (*P* < 0.01) during the treatment period (Figures 10A–D).

#### TBs-E Increase ROS Generation by HT-29 Cells

As illustrated in Figures 10E,F, fluorescence staining was performed to understand whether TBs-E regulates ROS expression in addition to its effects on cell antioxidant enzymes. A significant increase in ROS fluorescence intensity was produced by the HT-29 cells after the 48-h TBs-E treatment, which plays an important role in M5-EPS-induced apoptosis.

## Discussion

Dark tea is the second most popular tea in China. TBs have been studied as the main active ingredient in dark tea and they may have application prospects in the fields of medical care, food, and daily care. TBs form during the fermentation of dark tea, and they are complex with caffeine, polysaccharide conjugates, residual catechins, and other components in the tea. It is very difficult to purify and prepare free TBs from dark tea, which limits in-depth research, development, and applications. In this study, tea polyphenols were used as the raw materials to prepare TBs-E through enzymatic and chemical oxidation processes. The preparation was simple and environmentally friendly.

Several studies have focused on the anticancer activities of TBs, particularly inhibiting cancer cell proliferation, cell apoptosis, and cell cycle regulation through signaling pathways (20, 21). However, our study showed that TBs-E, mainly composed of extracted TBs inhibited proliferation and apoptosis of colon cancer cells and regulated the cell redox system. Cancer cells rely on a balance of antioxidant enzymes and ROS inside mitochondria (22). TBs-E acts as an exogenous stimulator to significantly enhance ROS expression and reduce enzyme activities, creating oxidative stress in the cell. Enzyme production was not sufficient to eliminate the soaring increase in ROS, and TBs-E reduced the antioxidant enzyme concentrations leading to the accumulation of ROS. Prolonged oxidative stress resulted in cell damage and death. These results will play an important role in promoting in-depth research, development, and industrialization of TBs.

## Data Availability Statement

The raw data supporting the conclusions of this article will be made available by the authors, without undue reservation.

## Author Contributions

XC: conceptualization, funding acquisition, investigation, methodology, project administration, supervision, and writing-review and editing. WS: writing-review and editing. ZW: resources. XL: validation. YY and YH: investigation and writing-original draft. BW and YC: data curation. WZ: formal analysis. All authors contributed to manuscript revision, read, and approved the submitted version.

## Funding

This study was financially supported by the National Natural Science Foundation of China (grant number 31871813) and the Beijing Advanced Innovation Center for Food Nutrition and Human Health.

## Conflict of Interest

The authors declare that the research was conducted in the absence of any commercial or financial relationships that could be construed as a potential conflict of interest.

## Publisher's Note

All claims expressed in this article are solely those of the authors and do not necessarily represent those of their affiliated organizations, or those of the publisher, the editors and the reviewers. Any product that may be evaluated in this article, or claim that may be made by its manufacturer, is not guaranteed or endorsed by the publisher.
